# Occurrence of Textile Dyes and Metals in Tunisian Textile Dyeing Effluent: Effects on Oxidative Stress Status and Histological Changes in Balb/c Mice

**DOI:** 10.3390/ijms222212568

**Published:** 2021-11-22

**Authors:** Nosra Methneni, Khawla Ezdini, Nouha Ben Abdeljelil, Joris Van Loco, Kathy Van den Houwe, Riheb Jabeur, Ons Fekih Sallem, Ahlem Jaziri, Mercedes Fernandez-Serrano, Nezar H. Khdary, Hedi Ben Mansour

**Affiliations:** 1 Research Unit of Analysis, Process Applied to the Environment–APAE (UR17ES32) Higher Institute of Applied Sciences and Technology Mahdia, University of Monastir, Monastir 5000, Tunisia; methneninosra28@gmail.com (N.M.); Onsfekihsallem@gmail.com (O.F.S.); a.jaziri12@gmail.com (A.J.); 2 Laboratory of Chemical Residues and Contaminants, Direction of Food Medicines and Consumer Safety, 1050 Brussels, Belgium; Joris.VanLoco@sciensano.be (J.V.L.); Kathy.VandenHouwe@sciensano.be (K.V.d.H.); 3 Department of Chemical Engineering, Faculty of Sciences, University of Granada, 18002 Granada, Spain; mferse@ugr.es; 4 Laboratory of Genetic, Biodiversity and Bio-Resources Valorisation, University of Monastir, Monastir 5000, Tunisia; ezzdinikhawla@gmail.com; 5 Department of Pathology, Fattouma Bourguiba University Hospital, Monastir 5000, Tunisia; nouhba@yahoo.fr; 6 Department of Matter and Life Sciences, Bretagne Sud University, IRDL, FRE CNRS 3744, CER Yves Coppens, BP573, 56000 Vannes, France; rihebjabeur1994@gmail.com; 7 King Abdulaziz City for Science and Technology (KACST), 11442 Riyadh, Saudi Arabia

**Keywords:** textile dyeing effluent, mice, oxidative stress, histopathology, metals, toxicity

## Abstract

Although it is known that textile wastewater contains highly toxic contaminants whose effects in humans represent public health problems in several countries, studies involving mammal species are scarce. This study was aimed to evaluate the toxicity profile of 90-days oral administration of textile dyeing effluent (TDE) on oxidative stress status and histological changes of male mice. The TDE was collected from the textile plant of Monastir, Tunisia and evaluated for the metals, aromatic amines, and textile dyes using analytical approaches. Metal analysis by ICP-MS showed that the tested TDE exhibited very high levels of Cr, As, and Sr, which exceeded the wastewater emission limits prescribed by WHO and Tunisian authority. The screening of TDE through UPLC-MS/MS confirmed the presence of two textile dyes: a triphenylmethane dye (Crystal violet) and a disperse azo dye (Disperse yellow 3). Exposure to TDE significantly altered the malondialdehyde (MDA), Conjugated dienes (CDs), Sulfhydryl proteins (SHP) and catalase levels in the hepatic and renal tissues. Furthermore, histopathology observation showed that hepatocellular and renal lesions were induced by TDE exposure. The present study concluded that TDE may involve induction of oxidative stress which ensues in pathological lesions in several vital organs suggesting its high toxicity. Metals and textile dyes may be associated with the observed toxicological effects of the TDE. These pollutants, which may have seeped into surrounding rivers in Monastir city, can cause severe health malaise in wildlife and humans.

## 1. Introduction

The textile industry is one of the major industrial polluting sectors of the environment and receiving water bodies, particularly in developing countries [1]. The wastewater generated by textile plants is very complex, since a large quantity and variety of chemicals are used during different textile operational stages.

Diverse types of chemical pollutants have been detected in textile wastewater, such as metals, textile dyes, surfactants, biocides, flame-retardants, and plasticizers [2,3,4]. Conventional wastewater treatment processes cannot effectively remove these toxic pollutants [4,5,6]. Furthermore, no standard limits exist for these pollutants and the physicochemical properties such as biochemical oxygen demand (BOD), chemical oxygen demand (COD), total phosphorus (TP), ammonia nitrogen, and total nitrogen (TN) are still adopted as the primary control standard for the release of industrial effluents from wastewater treatment companies.

It is documented that textile wastewater can induce many harmful effects, such as reprotoxicity, endocrine disruption, ecotoxicity, carcinogenicity, genotoxicity, and mutagenicity [7,8,9,10]. Several researchers have investigated the toxic effects of textile wastewater focusing mainly on the adverse effects to aquatic species such as algae, marine bacteria, daphnia, fish, and duckweed [11,12,13]. However, knowledge about the mixed toxicity of textile wastewater on mammal species is incomplete. No absolute criteria exist for opting a particular animal species in toxicological bioassays. To assess the acute and chronic toxicity of textile wastewater, rodents (mice and rats) have been extensively used [14,15,16]. However, these studies are limited to evaluating the toxicity of textile effluents based on conventional parameters such as body weight, organ weight, histopathology, and clinical chemistry. Little data are available on the assessment of the toxic effects of textile effluents using oxidative stress biomarkers. Biological tests in combination with chemical analysis may help immensely in deciphering toxicity as they express living organism response to the total effect of potential and actual disruption. 

In the developing world including Tunisia, the discharge of textile effluents into aquatic environments is the subject of discussion since these discharges are mostly made untreated or partially treated due to poor application of existing laws. For example, in Monastir state (latitude 35.60548/longitude 10.787695; located in the central-eastern of Tunisia), which houses more than 511 small-scale textile plants, a huge volume of textile wastewater is discharged. This is mostly untreated or poorly treated, and flows into the adjacent rivers. This disposal practice has affected the environmental quality of this region and caused a serious pollution problem [17]. The ecotoxicological effects of textile dyeing effluent (TDE) from the Monastir area were confirmed via a battery of bioassays with species belonging to different trophic levels (bacteria, algae, daphnia and plants) [13]. Nevertheless, much less attention is given to evaluating, in depth, the effect of these water matrices on mammals.

Therefore, the aim of the present study was to investigate the chronic toxic effects of untreated TDE on mice, as a complex mixture of chemical pollutants. TDE was subjected to chemical analysis for the determination of metal, aromatic amines and textile dyes contents. Male mice were exposed to different dilutions (25, 50 and 100%) of TDE. After 90 days exposure, oxidative stress, and histopathological studies were conducted. To our knowledge, this is the first report on the occurrence of chemical pollutants in TDE and their chronic effects on oxidative stress and histological changes in male mice.

## 2. Results

### 2.1. Occurrence of Metals, Textile Dyes, and Aromatic Amines

The concentration of metallic elements, textile dyes, and aromatic amines in the TDE has been illustrated in Table 1 and Table 2.

Metal quantification by the inductively coupled plasma mass spectrometry (ICP-MS) technique showed that the tested TDE exhibited a very high amount of Cr with a concentration of 0.05 mg/L and the value exceeded the wastewater emission limits into public sewers specified by Tunisian authority. The other target metals were below the Tunisian standard limits. Strontium was found to be the most abundant metal followed by manganese whereas Ag and Cd were not detected in the TDE. As and Sr were found to be in higher amounts than the wastewater emission limits suggested by the Word Health Organization (WHO) [3,18]. Out of the 10 textile dyes that were analyzed in the TDE through the Ultra-Performance Liquid Chromatography-tandem Mass Spectrometry (UPLC-MS/MS) technique, only the crystal violet and the disperse yellow 3 were detected with concentrations of 0.015 μg/L and 2.22 μg/L, respectively (Table 2). The target aromatic amines were not detected since their concentrations were below the limit of detection.

### 2.2. In Vivo Toxicological Investigations

#### 2.2.1. Biochemical Indicators of Lipid Peroxidation and Oxidative Stress

##### Malondialdehyde (MDA) Level in Homogenate of Liver and Kidney

**Figure 1:**
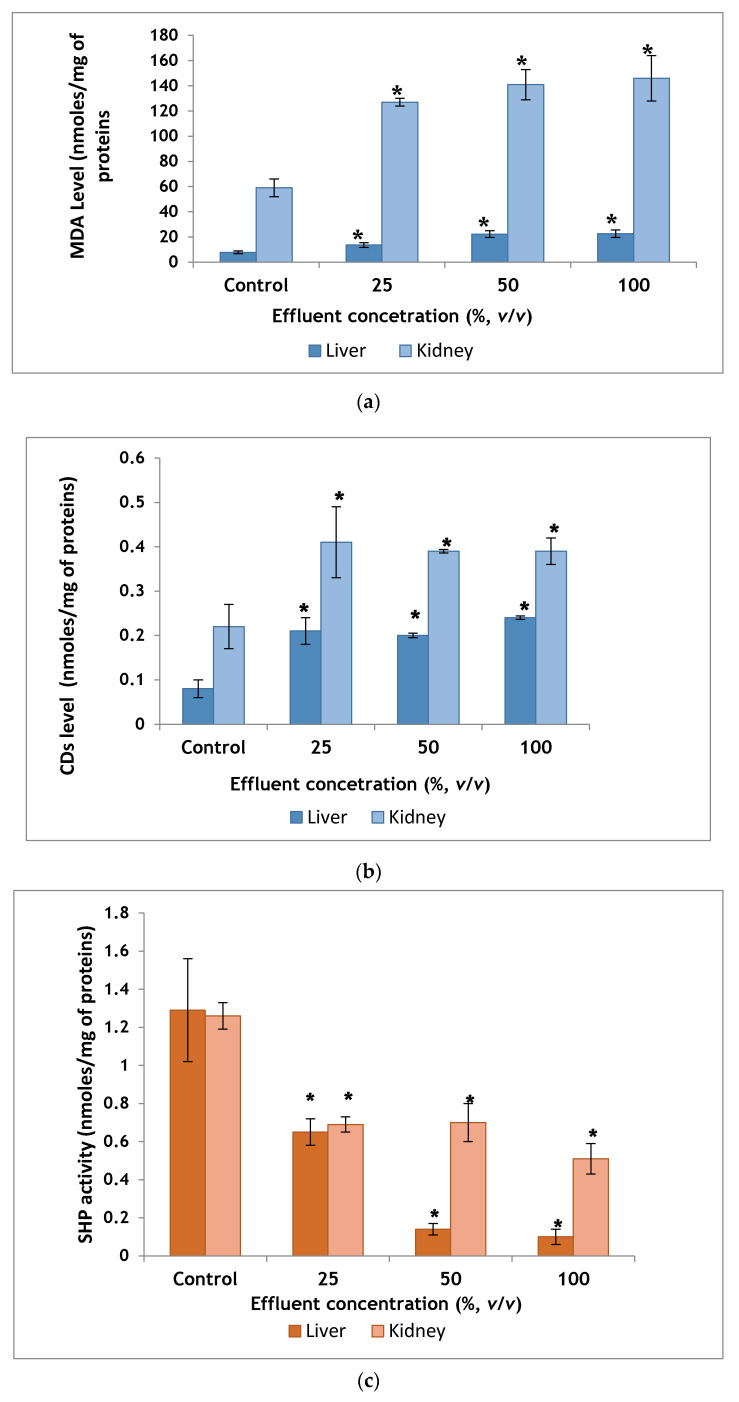

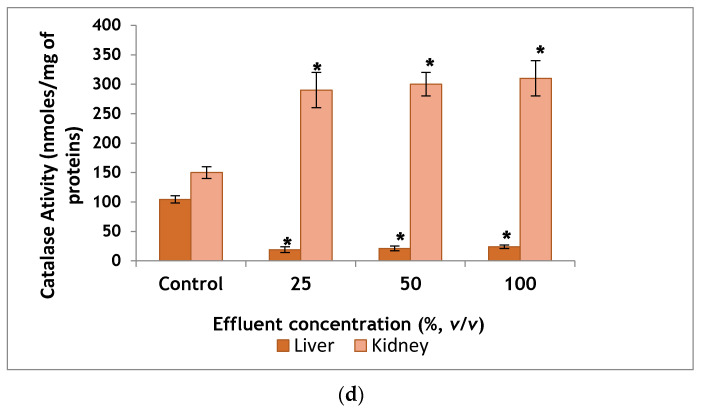
Results of the biochemical biomarkers [MDA level (**a**), CDs level (**b**) SHP activity (**c**), and catalase activity (**d**)] in the mice’s liver and kidney after 90 days exposure to various dilutions of TDE. Data are expressed as mean ± standard Deviation. Values are significantly different * *p* < 0.05 compared to the corresponding negative controls using one-way ANOVA followed by post hoc Tukey’s test.

Liver homogenates of the group receiving 100% TDE showed MDA level 22.6 ± 2.9 nmoles/mg of proteins, which was higher than the control group which exhibited MDA level of 7.76 ± 1.1 nmoles/mg of proteins and the difference was statistically significant (Figure 1a).

MDA concentration in kidney homogenates of control mice was found to be 59 ± 7 nmoles/mg of proteins. MDA concentration in treated groups showed an increase in MDA level and was significantly different when compared to the control group (Figure 1a).

##### Conjugated Dienes (CDs) Level in Homogenate of Liver and Kidney

Significant differences in terms of liver CDs level were observed among experimental groups, with a significant increase in mice exposed to the highest tested concentration relative to those from the control group (Figure 1b). 

Kidney homogenates of the group receiving 100% TDE showed CDs level 0.39 ± 0.03 nmoles/mg of proteins, which was higher than the control group, which exhibited CDs level of 0.22 ± 0.05 nmoles/mg of proteins and the difference was statistically significant (Figure 1b). 

##### Sulfhydryl Proteins (SHP) Activity in Homogenate of Liver and Kidney

SHP activity in liver and kidney homogenates of control mice was estimated to be 1.29 ± 0.27 and 1.26 ± 0.07 nmoles/mg of proteins, respectively whereas in group receiving 100% TDE was 0.1 ± 0.04 and 0.51 ± 0.08 nmoles/mg of proteins, respectively. SHP activity in liver and kidney homogenates was inversely proportional to TDE concentration (Figure 1c). 

##### Catalase activity in Homogenate of Liver and Kidney

Liver homogenates of the group receiving 100% TDE showed catalase activity 24 ± 3 nmoles/mg of proteins, which was 77% lower than control group, which exhibited catalase activity of 104.4 ± 6.2 nmoles/mg of proteins and the difference was statistically significant (Figure 1d).

The renal activity of catalase in the control group was 150 ± 10 nmoles/mg of proteins. In the group receiving 100% TDE, an activity of 310 ± 30 nmoles/mg of proteins was recorded. A significant increase was registered compared to the control mice (Figure 1d).

#### 2.2.2. Histopathological Assessment of the Liver and Kidney

**Figure 2:**
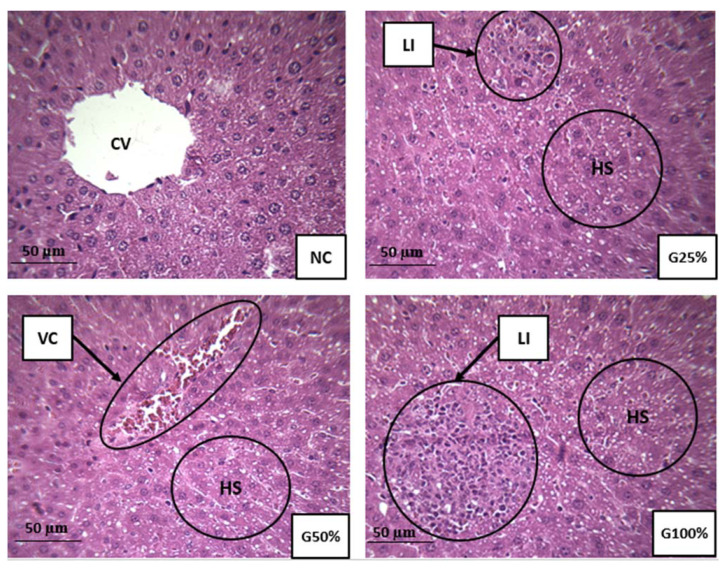
Histological sections of the liver from mice exposed to various dilutions of TDE (H&E, ×400). NC: liver section from negative control mice group showing apparently a healthy architecture of hepatocytes. G25%: liver sections of mice receiving 25% diluted TDE. G50%: liver sections of mice receiving 50% diluted TDE. G100%: liver sections of mice receiving 100% TDE. CV, central vein; LI, leucocyte infiltration; VC, vascular congestion of the central vein; HS, hepatic steatosis.

Figure 2 portrays the histopathological sections of the liver from TDE treated and control mice. Liver section from the negative control mice showed apparently normal hepatocytes with well-preserved cytoplasm and prominent nucleus. Exposure of mice to TDE led to different histological changes in the liver such as leukocyte infiltration, vascular congestion of the central vein, and hepatic steatosis.

**Figure 3:**
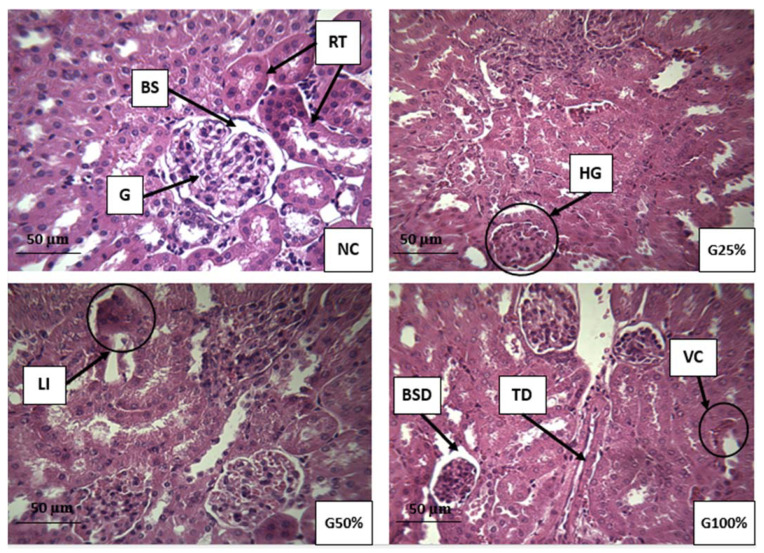
Histological sections of the kidney from mice exposed to various dilutions of TDE (H&E, ×400). NC: Kidney section from negative control mice group showing apparently normal structure of nephron’s the cortex and medulla. G25%: kidney section of mice receiving 25% diluted TDE. G50%: kidney section of mice receiving 50% diluted TDE. G100%: kidney section of mice receiving 100% TDE. G, glomerulus; BS, Bowman’s space; RT, renal tubules; VC, vascular congestion; LI, leucocyte infiltration; HG, hypertrophied glomerulus; BSD, Bowman’s space dilatation; TD, tubular dilatation.

Figure 3 portrays the histological sections of the kidney from TDE treated and control mice. Kidney sections from the negative control mice showed apparently normal histology of the cortex and medulla. Nevertheless, some nephrotoxic lesions were observed in the treated groups such as hypertrophied glomerulus, tubular dilatation, Bowman’s space dilatation, vascular congestion, and leukocyte infiltration.

## 3. Discussion

Complex industrial discharges such as textile wastewater contain high amounts of organic and inorganic pollutants, which pose serious risks to environmental health or ecosystems. Metal analysis by ICP-MS showed that the tested TDE exhibited a very high level of Cr, which exceeded the wastewater emission limits prescribed by Tunisian authority. As and Sr were found to be in elevated levels beyond the wastewater emission limits suggested by WHO [3,18]. The occurrence of different concentrations of metals has been well documented in TDE [3,19,20]. The presence of heavy metals in TDE is attributed to the use of metal complex dyes, stripping agents, mordents, chlorinated compounds, yarn waste, oxidizing agents, resins and organic solvents during different stages of textile processes [20,21]. Some of these metals present in the TDE persist in the environment, contaminate the surrounding water bodies and therefore pose ecotoxicological risks [22]. 

Azo and triphenylmethane dyes are extensively used by the textile sector in the dyeing process. Dyes are characterized by their persistence in aquatic environments due to their resistance to many types of treatment and difficulty of mineralization [23,24]. Therefore, when released into aquatic environments, wastewater containing these pollutants can induce toxic responses such as mutagenicity, genotoxicity, carcinogenicity and reprotoxicity [25,26,27]. The screening of TDE through UPLC-MS/MS confirmed the presence of two textile dyes: a triphenylmethane dye (crystal violet) and a disperse azo dye (disperse yellow 3). In contrast to our findings, several authors analyzed the presence of disperse yellow 3 in textile wastewater samples and found it undetectable [28,29]. This dye remaining in the TDE could alter ecosystems due to the discharge of large volumes of wastewater daily into nearby rivers. Recently, this same dye was detected with high levels in two surface water rivers located in Monastir city and highly contaminated by textile discharge [17]. Since microbes poorly metabolize it, crystal violet dye has been classified as a recalcitrant substance with a long lifespan in the environment. Conventional wastewater treatment techniques fail therefore to effectively remove crystal violet from wastewater resulting in its persistence in the environment. Chemical data related to the quantification of this dye in textile aqueous samples are relatively scarce. However, the occurrence of this dye and their metabolites in fish samples has been well documented [30,31]. Ref. [32] detected crystal violet in river sediments and soil that could be attributed to the discharge of improperly treated chemical wastes. Nevertheless, currently no regulatory thresholds exist for textile dyes in Tunisian standard to ensure the protection of aquatic biota or human against these toxic contaminants or their combined effects.

Aromatic amines are used mainly as precursors in the manufacture of dyes, persisting as impurities in the final product, and they can also be formed from the cleavage of dyes after their release into the environment [33]. Aromatic amines exhibiting genotoxic and mutagenic effects [34] remain of public health concern since they have been detected in clothing textiles [35] and environmental samples [36,37]. Target aromatic amines are not detected in the tested TDE. 

In the present study, the role of TDE was evaluated for induction of oxidative stress in mice. In this context, biochemical and histological markers in the livers and kidneys were analyzed. The liver and kidneys were selected since they are the first targeted organs by toxic damage. Hepatocytes contain a large number of metabolizing enzymes capable of biotransformating xenobiotic into less toxic or active metabolites. The liver is a lipid peroxidation site therefore considered a hallmark for oxidative stress tests. The kidneys perform the same functions along with filtration [38]. Histological analysis provides the most reliable data on the type of alterations induced by pollutants in tissues. Extremely toxic nature of TDE was evident from the histopathological alterations recorded in mice following chronic exposure. Although no death was recorded during the 90-day exposure period, severe damage to the liver and kidney was induced by hazardous textile wastewater. These findings are generally consistent with other studies [14,15,20,39]. These authors confirm that exposure to textile effluents leads to a disruption in the histo-architecture of kidney and liver tissues in rats and mice. The histological alterations observed in our study may be due to increased cellular formation of oxidative stress via the creation of an imbalance between the production of reactive oxygen species and cellular antioxidant capacities [40]. There is no universal marker for oxidative stress status. A critical point of standard biomarkers is the insufficient data on the mechanism of action of pollutants present in environmental samples using a single standard biomarker [41]. Therefore, the use of multiple biomarkers panel is recommended for the biomonitoring of environmental samples. The pro-oxidant nature of TDE was evident in our study since it deteriorated the antioxidant activities of enzymes in the liver and kidney of exposed mice. Lipid peroxidation is an oxidation reaction of unsaturated lipids mediated by free radicals and once initiated it is self-perpetuating. The length of the chain propagation depends on the antioxidant enzymes that break the chains [42]. Evaluation of the MDA and CDs contents, as degradation products of polyunsaturated fatty acids, are important indicators of lipid peroxidation. Our results indicated a significant elevation in MDA and CDs concentrations in hepatic and renal tissues as a result of TDE administration. The SHP activity is a valuable indicator of oxidative protein damage and of oxidative stress. Due to their ability to be easily oxidized, sulfhydryl groups are vulnerable to oxidative stress allowing the alteration of sulfhydryl-disulfide balance, which is a key factor in redox sensitive processes [43]. Our results showed a significant decrease in SHP contents in both tissues from mice exposed to TDE. Catalase activity constitutes an enzymatic defense system against radical aggression. The main function of this enzyme is the decomposition of hydrogen peroxide into molecular oxygen and water to protect tissues against aggression by highly reactive hydroxyl radicals [44]. The function of this enzyme may be affected during reactive oxygen species production leading to induction of DNA damage and pathological disorders [45]. In our study, the disruption of the enzymatic activity of catalase in treated mice suggests harmful effects of the constituents present in TDE via the excessive generation of non-detoxified free radicals. Similarly, these unscavenged free radicals induced the formation of lipoperoxide, which caused lipid peroxidation in the cell membrane of mice exposed to TDE compared to the negative control, which explains the increase in the MDA and CDs levels in the exposed mice to TDE. Previously, [19] reported that exposure to different dilutions of TDE for 60 days, was able to alter oxidative status markers, namely catalase activity, total superoxide dismutase activity and hydrogen peroxide level in liver, kidney and plasma of Wistar rats. Moreover, our findings showed that the recorded levels of MDA, CDs, SHP and catalase in the kidney were higher than those recorded in the liver. Therefore, the kidneys appear to be oxidatively more affected than the liver due to the administration of TDE. The extent of oxidative stress seems to be different in different organs, reflecting differences in reactive oxygen species production, metabolism, function, and distribution of endogenous antioxidant defenses.

The biochemical findings corroborate the observed histopathological lesions in the liver and kidney of mice exposed to various concentrations of TDE, suggesting liver and kidney dysfunctions induced by contaminants present in the wastewater. Two distinct chemical causes must be considered, with regard to the observed alterations in the livers and kidneys of mice following exposure to TDE: metals and textile dyes. Even at concentrations below the standard discharge limits, chemical pollutants can have deleterious effects on aquatic ecosystems and human health, once they are released into the environment. Various metals can generate the formation of free radicals causing lipid peroxidation and DNA damage leading to mutagenic and carcinogenic effects. Other metals can bind to the antioxidant glutathione and other proteins. Excess levels of As, Cr, and Cu increase the formation of reactive oxygen species through Fenton and Haber-Weiss oxidative reactions [46,47]. An excessive amount of As has also been associated with excessive generation of free radicals, induction of oxidative stress-related DNA damage, protein damage, and mutagenic, carcinogenic and cytotoxic effects [48]. As metal has been reported to induce oncogene amplification, cell transformation, genotoxicity, and carcinogenicity in humans [49]. The chronic exposure of cultured cells and rats to Cr, a widely found wastewater contaminant, may induce oxidative stress related apoptosis and alter the expression of tumor suppressor gene (p53) [50,51]. Chromium can also disrupt cell-signaling pathways, for example via the alteration of pyridoxine, which prevents oxidative damage linked to chromium [52]. An excess of Cu may alter oxidative defense system homeostasis leading to DNA damage, mutations and cytotoxicity [53]. Adverse DNA effects have been found experimentally following exposure to a dose of copper with as low as 15 µg/L [54]. Ref. [55] found that an excess of Zn in alga may cause oxidative stress related lipid peroxidation and alter cellular membrane permeability. Chronic exposure to a low dose of manganese may provoke DNA damage and increase the formation of dangerous free radicals [56].

Textile dyes are the second possible contributor to the observed toxicity in mice treated by TDE. There are no published studies on the ability of disperse yellow 3 and crystal violet to induce oxidative stress status. Several toxicological reports have revealed an association between both dyes and the incidence of mutagenicity and carcinogenicity [2,57]. In vitro and in vivo toxicological studies reported that crystal violet dye has been shown to be a mitotic poisoning agent and a biohazard substance [58]. Furthermore, crystal violet is considered to be a potent clastogenic agent leading to the promotion of tumor growth in certain aquatic organisms [57,59]. Since textile dyes are major contaminants of textile wastewater and represent a class of chemicals belonging to different groups with different chemical functions, the possible effects of their mixtures on mammals endorse further toxicological investigation. Nevertheless, it is important to point that TDE contains several unknown pollutants, which can exhibit a risk of unidentified extent. Indeed, their synergistic, antagonistic or additive interactions might alter the response to exposure. 

## 4. Materials and Methods

### 4.1. Wastewater Sampling

The TDE monitored in this study was sampled in 2018 from a textile industry located in Monastir, Tunisia (latitude 35.60548/longitude 10.787695). The mean wastewater flow is approximately 500 m^3^ /day, mainly discharged by cotton dyeing processes. During the day, chemicals released in the textile dyeing process are mixed in the swimming pools. Therefore, wastewater sampling is done in the morning (prior to initiation of dyeing processes on the next day) to ensure that the variability of the substances has been maintained in the analysis and to control their cumulative toxicity. Industrial effluent was transported to the laboratory, filtered with a 0.45 µm glass fiber Whatman filter (Sigma-Aldrich, Quimica, Belgium) and stored at −20 °C until chemical and biological testing.

### 4.2. Quantification of Metal Elements

The concentrations of metals such as (Li, Sc, Ti, Ag, Cd, V, Cr, Mn, Co, Ni, Cu, Zn, Ga, As, Se, Rb, Sr, Mo, Sn, Sb, Ba, Pb, and U) in the TDE were quantified through the ICP-MS technique (NexION 300X; Perkin Elmer, NY, USA) following the acid digestion of the textile wastewater. 

### 4.3. Analysis of Textile Dyes and Aromatic Amines 

The concentrations of 10 textile dyes belonging to different families, namely triphenylmethane dyes (Malachit green, Leuco-malachit green, Crystal violet, Leuco-crystal violet and Brilliant green), disperse azo dyes (Disperse Yellow 3, Disperse Orange 37 and Disperse Red 1) and sulfonated azo dyes (Acid Red 73 and Tartrazine), were determined in the wastewater sample using the UPLC-MS/MS method following [13,17].

A total of 25 aromatic amines (2,4-dimetylaniline, 3,3′dimetylbenzidine, Ortho-aminoazotoluene, 2,2-dichloro-4,4′-methylenedianiline, 4-aminoazobenzene, 3,3′dimethoxybenzidine, o-anisidine, 4,4′-diaminodiphenylether, Aniline, 4,4′-methylene-di-o-toluidine, 4-chloro-o-toluidine, 2-methoxy-5-methylaniline, 4-chloroaniline, p-phenylenediamine, 1,5-diaminonaphtalene, m-phenylenediamine, 3,3-dichlorobenzidine, 4,4′-thiodianiline, 2,4-diaminotoluene, o-toluidine, 2,6-dimetylaniline, 2,6-diaminotoluene, 2,4,5-trimethylaniline, 2-methyl-5-nitroaniline, 4,4′-diaminophenylmethane) were analyzed by UPLC-MS/MS [17].

The limit of detection was defined as the lowest level of the selected compound, which could be detected by UPLC-MS/MS. It was determined in our case through the signal-to-noise ratio of 3 following the validation method outlined previously [60]. 

### 4.4. In Vivo Study 

#### 4.4.1. Animals and Treatments 

A total of 24 healthy male Balb/c mice (30–35 g body weight, five to seven weeks old) were served as test animals and obtained from the animal facility of the central pharmacy of Tunisia (SIPHAT, Tunisia). Animals were acclimated one week before in vivo experiments in regulated environmental conditions (12-h light/dark photoperiod; temperature 25 ± 3 °C; humidity 40–60%) and fed a standard diet provided by SNA (SNA, Sfax, Tunisia) and tap water ad libitum. 25, 50, and 100% dilutions (*v*/*v*, TDE/distilled water) of the TDE were selected according to previous study [19]. Balb/c mice were randomly distributed into 4 groups (*n* = 6), 3 TDE-treated groups and a negative control group. Mice of each group were given individually free access to clean drinking water bottles and food ad libitum. For each mouse, the bottle was filled, weekly, with 28 mL of distilled water (negative control group) or TDE dilutions (treated groups). This research study was conducted for 90 days. The water volume was selected based on the average daily water consumption per mouse (4 mL) [61]. The mice exposure by free access to bottles was selected, since chronic administration by gavage can have potential confounding factors such as pulmonary injury, aspiration and induction of a stress response [62,63]. At post-exposure, animals were fasted for 24 h and then sacrificed by cervical dislocation. Samples of the liver and kidney were rapidly dissected out for oxidative stress markers analysis and histological study. The experimental protocol was approved by the animal ethics committee of the Pasteur Institute of Tunis (approval number: FST/LNFP/Pro 152012).

#### 4.4.2. Measurement of Oxidative Stress Biomarkers

Liver and kidney samples were homogenized (10% *w*/*v*) in ice-cold 10 mM phosphate buffer (pH 7.4) using a Silent Crusher M. type homogenizer (Heidolph Instruments GmbH & Co. KG, Schwabach, Germany). The homogenate was centrifuged at 12000 rpm for 10 min at 4 °C, and the resultant supernatant was stored at −80 °C and used for all subsequent assays.

Protein determination: Tissue protein concentration was determined using the method described by Bradford for protein estimation and calibrated using bovine serum albumin standard [64]. 

Catalase activity: The catalase activity was determined using a spectrophotometric method that monitors the decomposition of H_2_O_2_ at λ equal to 240 nm [65]. 

Estimation of MDA concentrations: To determine the extent of lipid peroxidation, the concentration of MDA was measured in accordance with [66]. MDA is known to form adducts with 2 thiobarbituric acid (TBA) molecules producing a pink colored pigment. Briefly, 100 µL of tissue homogenate supernatant was mixed with 1 ml of TBA and 900 µL of trichloroacetic acid (TCA). The reaction mixture was heated for 30 min in a boiling water bath. After cooling, the n-butanol was added and the mixture was mixed vigorously. After centrifugation of the samples (10000 rpm, 10 min), thiobarbituric acid reactive substances were determined by measuring the organic phase absorbance at 532 nm, and then expressed in terms of MDA formed.

Analysis of CDs levels: CDs level as indices of lipid peroxidation was assessed using the method outlined by [67]. The lipids were extracted with 2:1 (*v*/*v*) chloroform-methanol. The extract was evaporated into the oven (70 °C), then redissolved in hexane. The hexane solution was assayed at 243 nm. The CDs activity was expressed on µmoles/mg of protein.

SHP activity: Sulfhydryl contents of liver and kidney were quantified using the DTNB method [68]. Briefly, 50 µL of homogenate were mixed with 150 μL Tris (0.2 M; pH = 8.2), 40 μL EDTA (0.02 M) and 10 μL DTNB (0.01 M) and separated by centrifugation at 14500 rpm. A first absorbance measurement (OD1) was recorded at 412 nm and supernatants were supplemented with (5%) TCA for sulfhydryl protein precipitation. Then, 300 µL of the supernatant was incubated with 0.4 M tris and 0.01 M DTNB and a second absorbance measurement (OD2) was detected at 412 nm for non-sulfhydril proteins (N-SHP) analysis. SHP contents were calculated by subtracting the OD2 (containing the N-SHP) from the OD1 (containing the total proteins). Data was expressed as nmoles/mg of protein. 

Data assessment of oxidative stress biomarkers: the data of oxidative stress biomarkers was expressed as mean ± standard deviation and analyzed through one-way ANOVA. Differences among negative controls and treatment groups were determined using post hoc Tukey’s test using SPSS IBM 23 software. *p* values of less than 0.05 were considered statistically significant. 

#### 4.4.3. Histopathologycal Analysis

To evaluate the histopathological changes in the organs of effluent treated and control mice, livers and kidneys were removed and stored in 10% neutral buffered formalin. Histopathological investigations were performed using the standard procedures. In brief, the tissues were embedded in paraffin wax blocks, sectioned at 5-μm thick, mounted on glass slides and stained with hematoxylin-eosin (HE). Finally, the slides were examined at 400× magnification using a light microscope for any pathological alteration by a trained pathologist (a co-author on our publication) from the Department of Pathology, Fattouma Bourguiba University Hospital, Monastir, Tunisia. For histopathological assessment, six tissue samples for each group and three slices for each tissue were evaluated.

## 5. Conclusions

Textile wastewater contains a wide variety of toxic contaminants and may therefore be associated with a variety of toxic symptoms and chronic damage to living organisms. The mixed chronic effects of TDE was evaluated in this research using a combination of analytical and toxicological assays. Using the analytical approach, very high levels of Cr, As, and Sr, were detected in the tested TDE, which exceeded the wastewater emission limits prescribed by WHO and Tunisian authorities. A triphenylmethane dye (Crystal violet) and a disperse azo dye (Disperse yellow 3) were also detected. The toxicological approach revealed that after 90-day exposure period, TDE of Monastir city might be associated with generation of oxidative stress through alteration of MDA, CDs, SHP and catalase levels in the liver and kidney specimen of male mice and this stress may be attributed to the pathological lesions induced in these organs. It is suggested that these in vivo toxic effects were mediated by the metal elements and textile dyes present in the TDE. Further research is needed to assess the relationship between particulate pollutants present in TDE and the in vivo toxic effects, as well as the dose-response.

## Figures and Tables

**Table 1:** Concentrations of metals analyzed in the TDE sample and national and international permissible standards.

Metals	Concentrations (mg/L)	Tunisian Guide Level (mg/L)	WHO Guide Level (mg/L)
Li	0.07	NI	NI
Sc	0.001	NI	NI
Ti	0.003	NI	NI
Ag	ND	NI	NI
Cd	ND	NI	0.003
V	0.02	NI	NI
Cr	0.05	0.01	0.05
Mn	0.25	NI	0.5
Co	0.001	NI	NI
Ni	0.01	0.2	0.02
Cu	0.2	0.5	2
Zn	0.04	NI	2
Ga	0.004	NI	NI
As	0.02	0.05	0.01
Se	0.003	NI	NI
Rb	0.02	NI	NI
Sr	5.5	NI	0.05
Mo	0.005	NI	NI
Sn	0.004	2	NI
Sb	0.08	0.1	NI
Ba	0.14	NI	NI
Pb	0.001	NI	0.01
U	0.001	NI	NI

ND: not detected; WHO: World Health Organization; NI: not issued by the Tunisian and WHO guide levels.

**Table 2:** Concentrations of textile dyes analyzed in the TDE sample.

Target Compounds	Limit of Detection (μg/L)	Concentrations (μg/L)	Tunisian Guide Level
Malachit green	0.001	-	NI
Leuco-malachit green	0.001	-	NI
Crystal violet	0.001	0.015	NI
Leuco-crystal violet	0.001	-	NI
Brilliant green	0.001	-	NI
Disperse Yellow 3	0.002	2.22	NI
Disperse Orange 37	0.0136	-	NI
Disperse Red 1	0.0003	-	NI
Acid Red 73	0.001	-	NI
Tartrazine	0.006	-	NI

-: <limit of detection; NI: Not issued by Tunisian guide level.

## Data Availability

Data and Materials are available.

## References

[1] Savin I.L., Butnaru R. (2008). Wastewater characteristics in textile finishing mills. Environ. Eng. Manage. J..

[2] Carneiro P.A., Umbuzeiro G.A., Oliveira D.P., Zanoni M.V.B. (2010). Assessment of water contamination caused by a mutagenic textile effluent/dyehouse effluent bearing disperse dyes. J. Hazard. Mater..

[3] Akhtar M.F., Ashraf M., Javeed A., Anjum A.A., Sharif A., Saleem A., Akhtar B., Khan A.M., Altaf I. (2016). Toxicity appraisal of untreated dyeing industry wastewater based on chemical characterization and short term bioassays. Bull. Environ. Contam. Toxicol..

[4] Castro A.M., Nogueira V., Lopes I., Rocha-Santos T., Pereira R. (2019). Evaluation of the potential toxicity of effluents from the textile industry before and after treatment. Appl. Sci..

[5] Wille K., De Brabander H.F., De Wulf E., Van Caeter P., Janssen C.R., Vanhaecke L. (2012). Coupled chromatographic and mass-spectrometric techniques for the analysis of emerging pollutants in the aquatic environment. TrAC Trends Anal. Chem..

[6] Zocolo G.J., Pilon dos Santos G., Vendemiatti J., Vacchi F.I., de Aragão Umbuzeiro A., Zanoni M.V. (2015). Using SPE-LC-ESI-MS/MS analysis to assess disperse dyes in environmental water samples. J. Chromatogr. Sci..

[7] Suryavathi V., Sharma S., Sharma S., Saxena P., Pandey S., Grover R., Kumar S., Sharma K.P. (2005). Acute toxicity of textile dye wastewaters (untreated and treated) of Sanganar on male reproductive systems of albino rats and mice. Reprod. Toxicol..

[8] Lima R.O.A., Bazo A.P., Salvadori D.M.F., Rech C.M., Oliveira D.P., Umbuzeiro G.A. (2007). Mutagenic and carcinogenic potentiel of a textile azo dye processing plant effluent that impacts a drinking water source. Mutat. Res..

[9] Mansour H.B., Houas I., Montassar F., Ghedira K., Barillier D., Mosrati R., Chekir-Ghedira L. (2012). Alteration of in vitro and acute in vivo toxicity of textile dyeing wastewater after chemical and biological remediation. Environ. Sci. Pollut. Res..

[10] Schiliro T., Porfido A., Spina F., Varese G.C., Gilli G. (2012). Oestrogenic activity of a textile industrial wastewater treatment plant effluent evaluated by the *E-screen* test and MELN gene-reporter luciferase assay. Sci. Total Environ..

[11] Sharma K.P., Sharma S., Sharma S., Singh P.K., Kumar S., Grover R., Sharma P.K. (2007). A comparative study on characterization of textile wastewaters (untreated and treated) toxicity by chemical and biological tests. Chemosphere.

[12] Liang J., Ning X., Sun J., Song J., Lu J., Cai H., Hong Y. (2018). Toxicity evaluation of textile dyeing effluent and its possible relationship with chemical oxygen demand. Ecotox. Environ. Saf..

[13] Methneni N., González J.A.M., Jaziri A., Mansour H.B., Fernandez-Serrano M. (2021). Persistent organic and inorganic pollutants in the effluents from the textile dyeing industries: Ecotoxicology appraisal via a battery of biotests. Environ. Res..

[14] Oloyede A.M., Ogunlaja O., Ogunlaja A. (2014). Sub-chronic Toxicity assessment of local textile ‘Adire and Kampala’ (Tie and Dye) Effluents on Mice (*Mus musculus*). Res. J. Environ. Sci..

[15] Bhavesh K.V., Tank S.K. (2015). Toxicity study of textile effluent of Udhna, Surat Region (Gujarat) on wistar albino rat. Univers. J. Environ. Res. Technol..

[16] Afsa S., Sallem O.F., Abdeljelil N.B., Feriani A., Najjar M.F., Mansour H.B. (2021). In vivo toxicities of the hospital effluent in Mahdia Tunisia. J. Water Health.

[17] Methneni N., González J.A.M., Van Loco J., Anthonissen R., Van de Maele J., Verschaeve L., Fernandez-Serrano M., Mansour H.B. (2020). Ecotoxicity profile of heavily contaminated surface water of two rivers in Tunisia. Environ. Toxicol. Pharmacol..

[18] Shakir L., Ejaz S., Ashraf M., Ahmad N., Javeed A. (2012). Characterization of tannery effluent wastewater by proton-induced X-ray emission (PIXE) analysis to investigate their role in water pollution. Environ. Sci. Pollut. Res..

[19] Akhtar M.F., Ashraf M., Anjum A.A., Javeed A., Sharif A., Saleem A., Akhtar B. (2016). Textile industrial effluent induces mutagenicity and oxidative DNA damage and exploits oxidative stress biomarkers in rats. Environ. Toxicol. Pharmacol..

[20] Akhtar M.F., Ashraf M., Javeed A., Anjum A.A., Sharif A., Saleem A., Mustafa G., Ashraf M., Saleem A., Akhtar B. (2018). Association of textile industry effluent with mutagenicity and its toxic health implications upon acute and sub-chronic exposure. Environ. Monit. Assess..

[21] Zeiner M., Rezic I., Steffan I. (2007). Analytical methods for the determination of heavy metals in the textile industry. J. Chem. Chem. Eng..

[22] Hemachandra C., Pathiratne A. (2015). Assessing toxicity of copper, cadmium and chromium levels relevant to discharge limits of industrial effluents into inland surface waters using common onion, Allium cepa bioassay. Bull. Environ. Contam. Toxicol..

[23] Almeida E.J.R., Corso C.R. (2019). Decolorization and removal of toxicity of textile azo dyes using fungal biomass pelletized. Int. J. Environ. Sci. Technol..

[24] Daneshvar N., Ayazloo M., Khataee A.R., Pourhassan M. (2007). Biological decolorization of dye solution containing Malachite Green by microalgae *Cosmarium* sp. Bioresour. Technol..

[25] Jadhav J.P., Govindwar S.P. (2006). Biotransformation of malachite green by *Saccharomyces cerevisiae* MTCC 463. Yeast.

[26] Mansour H.B., Corroler D., Barillier D., Ghedira K., Chekir L., Mosrati R. (2007). Evaluation of genotoxicity and pro-oxidant effect of the azo dyes: Acids yellow 17, violet 7 and orange 52, and of their degradation products by *Pseudomonas putida* mt-2. Food Chem. Toxicol..

[27] Liu H., Yu H., Giesy J.P., Sun Y., Wang X. (2007). Toxicity of HC Orange No. 1 to *Daphnia magna*, zebrafish (*Brachydanio rerio*) embryos, and goldfish (*Carassius auratus*). Chemosphere.

[28] Vacchi F.I., Vendemiatti J.A., Brosselin V., da Silva B.F., Zanoni M.V.B., DeMeo M., Bony S., Devaux A., Umbuzeiro G.A. (2016). Combining different assays and chemical analysis to characterize the genotoxicity of waters impacted by textile discharges. Environ. Mol. Mutagen..

[29] Vacchi F.I., Vendemiatti J.A.S., da Silva B.F., Zanoni M.V.B., Umbuzeiro G.A. (2017). Quantifying the contribution of dyes to the mutagenicity of waters under the influence of textile activities. Sci. Total Environ..

[30] Schuetze A., Heberer T., Juergensen S. (2008). Occurrence of residues of the veterinary crystal (gentian) violet in wild eels caught downstream from municipal sewage treatment plants. Environ. Chem..

[31] Belpaire C., Reyns T., Geeraerts C., Van Loco J. (2015). Toxic textile dyes accumulate in wild European eel *Anguilla anguilla*. Chemosphere.

[32] Nelson C.R., Hites R.A. (1980). Aromatic amines in and near the Buffalo River. Environ. Sci. Technol..

[33] Freeman H.S. (2013). Aromatic amines: Use in azo dye chemistry. Front. Biosci..

[34] Josephy P.D., Zahid M., Dhanoa J., de Souza G.B.D., Groom H., Lambie M. (2016). Potent mutagenicity in the Ames test of 2-cyano-4- nitroaniline and 2,6-dicyano-4-nitroaniline, components of disperse dyes. Environ. Mol. Mutagen..

[35] Bruschweiler B.J., Kung S., Burgi D., Muralt L., Nyfeler E. (2014). Identification of non-regulated aromatic amines of toxicological concern which can be cleaved from azo dyes used in clothing textiles. Regul. Toxicol. Pharmacol..

[36] Oliveira D.P., Carneiro P.A., Sakagami M.K., Zanoni M.V.B., Umbuzeiro G.A. (2007). Chemical characterization of a dye processing plant effluent—Identification of the mutagenic components. Mutat. Res. Genet. Toxicol. Environ. Mutagen..

[37] Özkana B.C., Fırat M., Chormey D.S., Bakırdere S. (2019). Accurate and sensitive determination of harmful aromatic amine products of azo dyes in wastewater and textile samples by GC–MS after multivariate optimization of binary solvent dispersive liquid-liquid microextraction. Microchem. J..

[38] Patlolla A.K., Barnes C., Yedjou C., Velma V., Tchounwou P.B. (2009). Oxidative stress, DNA damage, and antioxidant enzyme activity induced by hexavalent chromium in Sprague-Dawley rats. Environ. Toxicol..

[39] Amin T., Afrin M., Haque Z., Islam M.R. (2016). Toxicity of textile dye wastewater on liver of mice. J. Agric. Vet. Sci..

[40] Adeoye G.O., Alimba C.G., Oyeleke O.B. (2015). The genotoxicity and systemic toxicity of a pharmaceutical effluent in Wistar rats may involve oxidative stress induction. Toxicol. Rep..

[41] Maselli B.D.S., Luna L.A.V., Palmeira J.D.O., Tavares K.P., Barbosa S., Beijo L.A., Umbuzeiro G.A., Kummrow F. (2015). Ecotoxicity of raw and treated effluents generated by a veterinary pharmaceutical company: A comparison of the sensitivities of different standardized tests. Ecotoxicology.

[42] Perluigi M., Coccia R., Butterfield D.A. (2012). 4-Hydroxy-2-Nonenal, a reactive product of lipid peroxidation, and neurodegenerative diseases: A toxic combination illuminated by redox proteomics studies. Antioxid. Redox Signal..

[43] Winterboum C.C., Hampton M.B. (2008). Thiol chemistry and specificity in redox signaling. Free Radic. Biol. Med..

[44] Timbrel J.A. (2009). Principles of Biochemical Toxicology.

[45] Ramaiah S., Jaeschke H. (2007). Role of neutrophils in the pathogenesis of acute inflammatory liver injury. Toxicol. Pathol..

[46] Valko M., Morris H., Cronin M. (2005). Metals, toxicity and oxidative stress. Curr. Med. Chem..

[47] Jomova K., Valko M. (2011). Advances in metal-induced oxidative stress and human disease. Toxicology.

[48] Liu S.X., Athar M., Lippai I., Waldren C., Hei T.K. (2001). Induction of oxyradicals by arsenic: Implication for mechanism of genotoxicity. Proc. Natl. Acad. Sci. USA.

[49] Dong J.-T., Luo X.-M. (1993). Arsenic-induced DNA-strand breaks associated with DNA-protein crosslinks in human fetal lung fibroblasts. Mutat. Res. Lett..

[50] Bagchi D., Bagchi M., Stohs S.J. (2001). Chromium (VI)-induced oxidative stress, apoptotic cell death and modulation of p53 tumor suppressor gene. Mol. Cell. Biochem..

[51] Bagchi D., Joshi S., Bagchi M., Balmoori J., Benner E., Kuszynski C., Stohs S. (2000). Cadmium-and chromium-induced oxidative stress, DNA damage, and apoptotic cell death in cultured human chronic myelogenous leukemic K562 cells, promyelocytic leukemic HL-60 cells, and normal human peripheral blood mononuclear cells. J. Biochem. Mol. Toxicol..

[52] Ezaka E., Anyanwu C. (2011). Chromium (VI) tolerance of bacterial strains isolated from sewage oxidation ditch. Int. J. Environ. Sci..

[53] Lee D.H., O’Connor T.R., Pfeifer G.P. (2002). Oxidative DNA damage induced by copper and hydrogen peroxide promotes CG→TT tandem mutations at methylated CpG dinucleotides in nucleotide excision repair-deficient cells. Nucleic Acids Res..

[54] Atienzar F.A., Cheung V.V., Jha A.N., Depledge M.H. (2001). Fitness parameters and DNA effects are sensitive indicators of copper-induced toxicity in *Daphnia magna*. Toxicol. Sci..

[55] Tripathi B.N., Gaur J. (2004). Relationship between copper-and zinc-induced oxidative stress and proline accumulation in *Scenedesmus* sp. Planta.

[56] Lima P.D.L., Vasconcellos M.C., Bahia M.O., Montenegro R.C., Pessoa C.O., Costa-Lotufo L.V., Moraes M.O., Burbano R.R. (2008). Genotoxic and cytotoxic effects of manganese chloride in cultured human lymphocytes treated in different phases of cell cycle. Toxicol. In Vitro.

[57] Mani S., Bharagava R.N. (2016). Exposure to crystal violet, its toxic, genotoxic and carcinogenic effects on environment and its degradation and detoxification for environmental safety. Rev. Environ. Contam. Toxicol..

[58] Parshetti G.K., Parshetti S.G., Telke A.A., Kalyani D.C., Doong R.A., Govindwar S.P. (2011). Biodegradation of crystal violet by *Agrobacterium* radiobacter. J. Environ. Sci..

[59] Fan H.J., Huang S.T., Chung W.H., Jan J.L., Lin W.Y., Chen C.C. (2009). Degradation pathways of crystal violet by fenton and fenton-like systems: Condition optimization and intermediate separation and identification. J. Hazard. Mater..

[60] Reyns T., Belpaire C., Geeraerts C., Van Loco J. (2015). Multi-dye residue analysis of triarylmethane, xanthene, phenothiazine and phenoxazine dyes in fish tissues by ultra-performance liquid chromatography-tandem mass spectrometry. J. Chromatogr. B.

[61] Williams O. (1959). Water intake in the deer mouse. J. Mammal..

[62] Siqueira I.R., Vanzella C., Bianchetti P., Rodrigues M.A.S., Stülp S. (2011). Anxiety-like behaviour in mice exposed to tannery wastewater: The effect of photoelectrooxidation treatment. Neurotoxicol. Teratol..

[63] Moysés F., Bertoldi K., Spindler C., Sanches E.F., Elsner V.R., Rodrigues M.A.S., Siqueira I.R. (2014). Exposition to tannery wastewater did not alter behavioral and biochemical parameters in Wistar rats. Physiol. Behav..

[64] Bradford M.M. (1976). A rapid and sensitive method for the quantitation of microgram quantities of protein utilizing the principle of protein-dye binding. Anal. Biochem..

[65] Zhang Y., Luo Y., Hou Y.X., Jiang H., Chen Q., Tang H.R. (2008). Chilling acclimation induced changes in the distribution of H_2_O_2_ and antioxidant system of strawberry leaves. Agric. J..

[66] Tabrez S., Ahmad M. (2009). Effect of wastewater intake on antioxidant and marker enzymes of tissue damage in rat tissues: Implications for the use of biochemical markers. Food Chem. Toxicol..

[67] Şahin E., Gümüşlü S. (2007). Immobilization stress in rat tissues: Alterations in protein oxidation, lipid peroxidation and antioxidant defense system. Comp. Biochem. Physiol. Part C Toxicol. Pharmacol..

[68] Manda K., Ueno M., Moritake T., Anzai K. (2007). Radiation-induced cognitive dysfunction and cerebellar oxidative stress in mice: Protective effect of ⍺-lipoic acid. Behav. Brain Res..

